# Description of two new species of the leafhopper subgenus *Pediopsoides* (*Pediopsoides*) (Hemiptera, Cicadellidae, Macropsinae) from Guangxi Province, Southern China

**DOI:** 10.3897/zookeys.321.5454

**Published:** 2013-08-02

**Authors:** Hu Li, Ren-Huai Dai, Zi-Zhong Li

**Affiliations:** 1 Institute of Entomology, Guizhou University, Guiyang, Guizhou, P.R. China, 550025; 2 The Provincial Key Laboratory for Agricultural Pest Management of Mountainous Region, Guiyang, Guizhou, P.R. China, 550025

**Keywords:** Auchenorrhyncha, taxonomy, morphology, description

## Abstract

Two new species of the Macropsinae leafhopper subgenus *Pediopsoides (Pediopsoides)* Matsumura, 1912, *Pediopsoides (Pediopsoides) damingshanensis* Li, Dai & Li, **sp. n.** and *Pediopsoides (Pediopsoides) tishetshkini* Li, Dai & Li **sp. n.**, are described and illustrated from Guangxi Province of southern China. A key to males is provided to distinguish the species of the subgenus along with a map showing the distribution of the new species.

## Introduction

The leafhopper genus *Pediopsoides* (Insecta: Auchenorrhyncha: Membracoidea: Cicadellidae: Macropsinae) was established by Matsumura (1912) for a single species, *Pediopsoides formosanus* Matsumura, 1912, from Taiwan island of China. In Hamilton’s (1980) Macropsini revision the genus *Pediopsoides* included 5 subgenera, including the nominate subgenus *Pediopsoides (Pediopsoides)* for *Pediopsoides (Pediopsoides) formosanus* Matsumura, 1912 from Taiwan island of China and *Pediopsoides (Pediopsoides) satsumensis* (Matsumura, 1912) from Kiushu island of Japan. More recently, the following other species have been added: *Pediopsoides (Pediopsoides) femorata* (Hamilton, 1980) from Taiwan island of China, *Pediopsoides (Pediopsoides) kodaiana* Viraktamath, 1996 from Tamil Nadu state of India, *Pediopsoides (Pediopsoides) jingdongensis* Zhang, 2010 from Yunnan Province of China, *Pediopsoides (Pediopsoides) bispinata* Li, Dai & Li, 2012 and *Pediopsoides (Pediopsoides) nigrolabium* Li, Dai & Li, 2012 from Guangxi Province of China. Until now there are seven species of the nominate subgenus including five from China.

In the present paper, 2 new species, *Pediopsoides (Pediopsoides) damingshanensis* Li, Dai & Li sp. n. and *Pediopsoides (Pediopsoides) tishetshkini* Li, Dai & Li sp. n., from Guangxi province (included in the oriental region) of southern China are described and illustrated, a key to species of the nominate subgenus are provided, along with a map showing the distribution of the new species.

## Material and methods

Terminology used in describing the structures follows Hamilton (1980).

External morphology and dissected male genitalia were observed under an Olympus SZ2-ILST stereoscopic microscope and YS 100 microscope. Habitus images of adults were obtained by using a KEYENCE VHX-1000 system and were processed using the software Adobe Photoshop CS6. Hand drawings of the male genitalia were processed with Adobe Illustrator CS6. The body length is measured from the apex of the head to the apex of the forewings and are given in millimeter (mm.).

The type specimens of the new species are all deposited in the Institute of Entomology, Guizhou University, Guiyang, China (GUGC).

## Taxonomy

### Subgenus Pediopsoides (Pediopsoides)

Matsumura

Pediopsoides Matsumura, 1912: 305.
Pediopsoides (Pediopsoides) Hamilton, 1980: 896.

#### Type species.

*Pediopsoides formosanus* Matsumura, 1912, by original designation.

#### Remarks.

The nominate subgenus was characterized by Hamilton (1980) and Li et al. (2012). It can be distinguished by the following combination of features: pronotal striations oblique, forewings with two anteapical cells, dorsal connective freely attached (fused in subgenus *Pediopsoides (Celopsis)*) to tenth tergite, and male pygofer without multifid or additional spines [male pygofer spine bifid or with rounded lobe on inner margin basally in subgenus *Pediopsoides (Sispocnis)*, a small secondary pygofer spine posteriorly in subgenus *Pediopsoides (Nanopsis)*, and male pygofer spine are multifid in subgenus *Pediopsoides (Kiamoncopsis)*].

#### Distribution.

Oriental region: China, Japan and India.

### Pediopsoides (Pediopsoides) damingshanensis

Li, Dai & Li sp. n.

http://zoobank.org/8874F63A-824B-43CC-9509-F1D6180A8B5E

http://species-id.net/wiki/Pediopsoides_damingshanensis

Figures 1–3 , 7–13 , 22

#### Type locality.

CHINA: Guangxi Province, Damingshan.

#### Measurements.

Body length (including tegmina), ♂, 4.0 mm.

#### Description.

**Figures 1–6.**
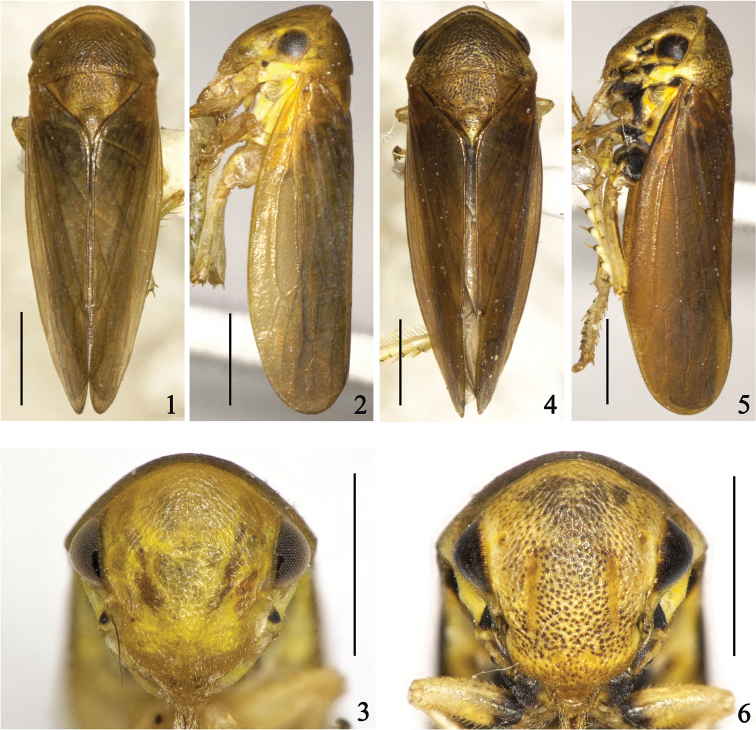
**1–3** *Pediopsoides (Pediopsoides) damingshanensis* Li, Dai & Li sp. n. **4–6** *Pediopsoides (Pediopsoides) tishetshkini* Li, Dai & Li sp. n. **1, 4** Dorsal habitus, male **2, 5** Lateral habitus, male **3, 6** Face.

*Body coloration*. General color (Figs 1–3) yellowish brown. Face (Fig. 3) yellowish with slight greenish tinge, eyes pale brown, slight brown oblique band adjacent inner sides of ocelli, lower part of face pale brown. Pronotum (Fig. 1) dark brown centrally, lateral and front margins gradually more yellowish. Scutellum (Fig. 1) dark brown except lateral margins yellowish. Forewings (Fig. 2) evenly dark brown except yellowish basal part. Legs pale yellowish.

*External morphology*. Head, face, pronotum, and scutellum faintly striated. Head (Fig. 1), in dorsal view, clearly arcuate forward, slightly narrower than pronotum, crown distinctly shorter medially than next to eyes. Face (Fig. 3), in lateral view, moderately flat; in facial view, as wide as long across eyes; dorsal part of frontoclypeus with slight medial longitudinal carina; ocelli about 9 times their diameter to adjacent eye; lacking clear sutures between lora and frontoclypeus. Pronotum (Fig. 1) 2.2 times as long as wide, frontally and laterally with oblique striations; anterior margin strongly arched, posterior margin excavate medially. Scutellum (Fig. 1) 1.3 times longer than pronotum. Forewings (Fig. 2) hyaline, with 2 anteapical cells, venation protruding. Hind femoral macrosetae 2+1; hind tibia with 7 macrosetae on AD row, 5 on AV row, 11–12 on PD row, dense and slender on PV row.

**Figures 7–13.**
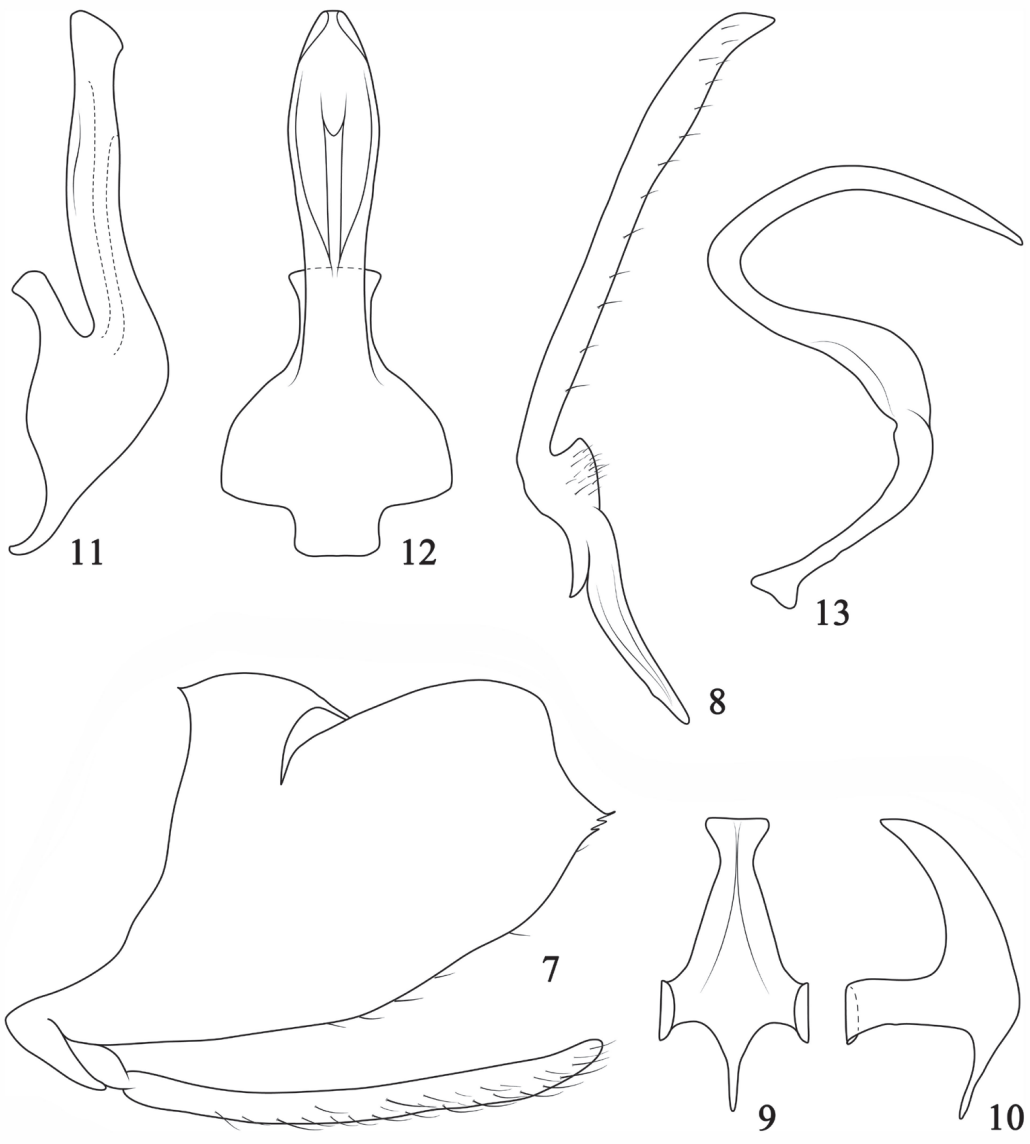
*Pediopsoides (Pediopsoides) damingshanensis* Li, Dai & Li sp. n. **7** Male pygofer side and subgenital plate, lateral view **8** Style, dorsal view **9** Connective, dorsal view **10** Connective, lateral view **11** Aedeagus, lateral view **12** Aedeagus, ventral view **13** Dorsal connective, lateral view.

*Male genitalia*. Pygofer (Fig. 7), with dorsal margin incised, caudal margin truncate, slightly sinuated in lateral view, ventral margin serrate distally with few fine setae. Subgenital plate (Fig. 7), in lateral view, slightly shorter than pygofer, slender, rod-like, with many scattered setae. Style (Fig. 8), slender, apophysis margined with short fine setae, slightly angled after lateral lobe, slightly inflated subapically, apex subacute. Connective (Figs 9–10) distinctly longer than greatest width basally, with anterior medial process long, arms bent dorsally. Aedeagus (Figs 11–12), with short preatrium; dorsal apodeme moderately long; shaft sinuate, in lateral view apex truncate, in ventral view broad basally, widened subapically; gonopore long, apical on ventral margin. Dorsal connective (Fig. 13) “S” shaped with acute apex.

*Female*. Unknown.

#### Type material.

Holotype, ♂, CHINA: Guangxi Province, Damingshan National Natural Reserve, 14. V. 2012, collected by Li Hu.

**Distribution.** Guangxi Prov. (Damingshan), China (Fig. 22).

#### Diagnosis.

This new species differs from other members of the subgenus *Pediopsoides (Pediopsoides)* by the shape of the male genitalia.

#### Etymology.

The new species name refers to the type locality, *Damingshan*.

### Pediopsoides (Pediopsoides) tishetshkini

Li, Dai & Li sp. n.

http://zoobank.org/9A8FCAC5-D112-43BC-A401-367DCDCE83E9

http://species-id.net/wiki/Pediopsoides_tishetshkini

Figures 4–6 , 14 –22

#### Type locality.

CHINA: Guangxi Province, Tianlin.

#### Measurements.

Body length (including tegmina), ♂, 4.5 mm.

#### Description.

*Body coloration*. Color (Figs 4–6) similar to *Pediopsoides (Pediopsoides) damingshanensis* but darker; with large black spot adjacent antennal pit and basal parts of fore femur and coxa, middle and hind coxa marked with dark brown.

*External morphology*. As in *Pediopsoides (Pediopsoides) damingshanensis* but head more narrower than pronotum and crown more arcuate forward; face (Fig. 6), in lateral view, slightly inflated; ocelli with their spacing about 8 times than that of ocellus to adjacent eye; scutellum (Fig. 4) about 1.4 times longer than pronotum. Hind femoral macrosetae 2+1; hind tibia with 7 macrosetae on AD row, 6 on AV row, 11 on PD row, dense and slender on PV row.

**Figures 14–21.**
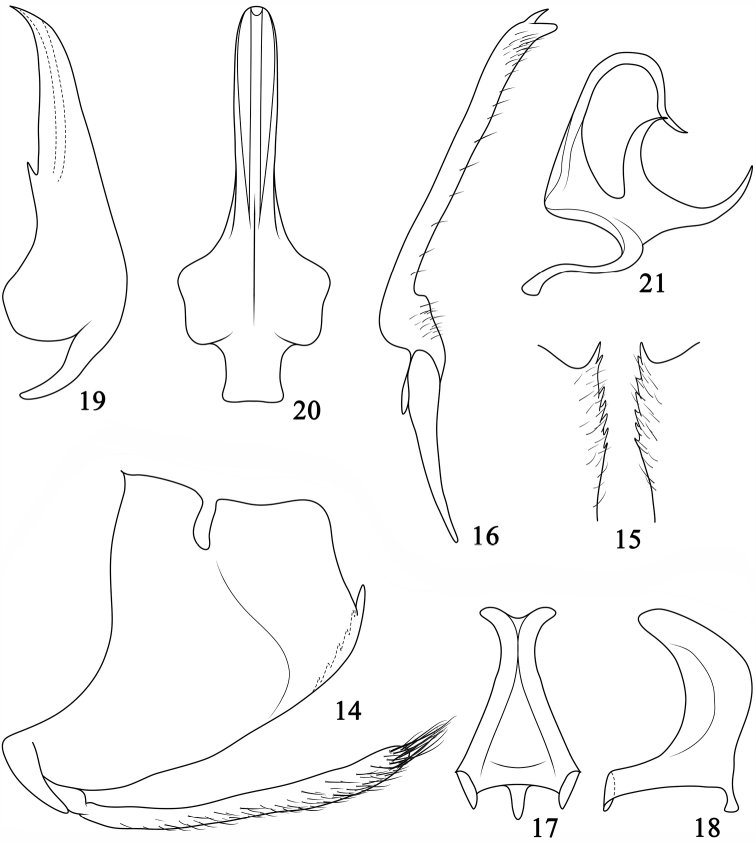
*Pediopsoides (Pediopsoides) tishetshkini* Li, Dai & Li sp. n. **14** Male pygofer side and subgenital plate, lateral view **15** Ventral margins of male pygofer **16** Style, dorsal view **17** Connective, dorsal view **18** Connective, lateral view **19** Aedeagus, lateral view **20** Aedeagus, ventral view **21** Dorsal connective, lateral view.

*Male genitalia*. Similar to *Pediopsoides (Pediopsoides) damingshanensis* but pygofer (Fig. 14) shorter, style (Fig. 16) with lateral lobe shorter and apex bearing a spine-like process and connective (Figs 17–18) more robust in lateral view. Aedeagus (Figs 19–20) with basal apodeme reduced; preatrium moderately long; shaft in lateral view relatively straight, evenly tapered to sharply pointed and upturned apex; in ventral view shaft similar in width throughout length with rounded apex; gonopore apical on ventral margin. Dorsal connective (Fig. 21) strongly developed “S” shaped, medially produced into bifurcate process, two dorsal branches widely spaced and tapered to acute apex.

*Female*. Unknown.

#### Type material.

Holotype, ♂, CHINA: Guangxi Province, Baise City, Tianlin County, Langping Village, 23. IV. 2012, collected by Zheng Weibin.

#### Distribution.

**Figure 22.**
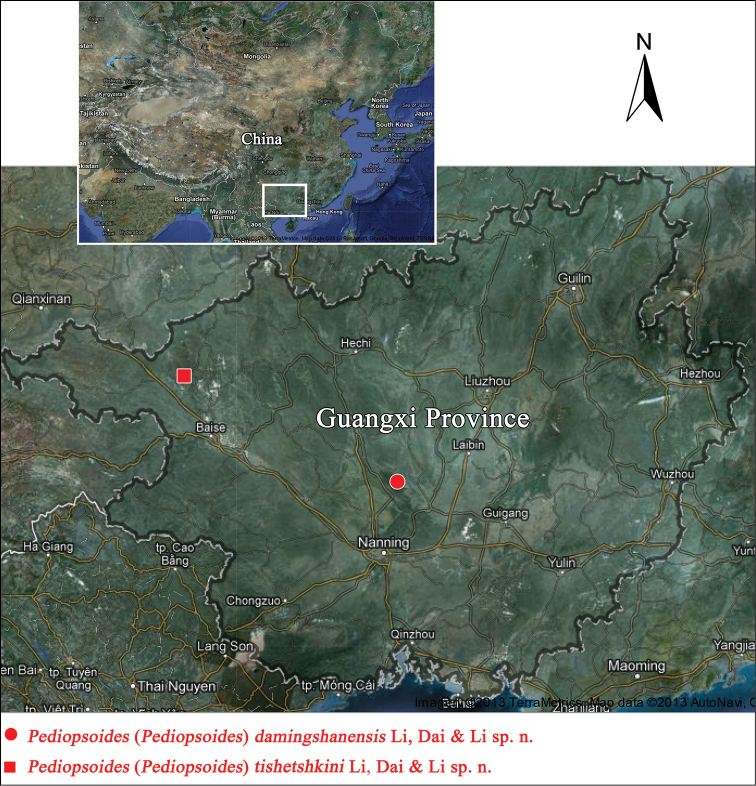
Map showing the distribution of *Pediopsoides (Pediopsoides) damingshanensis* Li, Dai & Li sp. n.and *Pediopsoides (Pediopsoides) tishetshkini* Li, Dai & Li sp. n.

Guangxi Prov. (Tianlin), China (Fig. 22).

#### Remarks.

The new species is similar to *Pediopsoides (Pediopsoides) damingshanensis* sp. n. but can be distinguished by its darker colour and differences in the male genitalia (see description).

#### Etymology.

This species is named in honour of Dr. Dmitri Yu. Tishechkin for his excellent contribution to *Macropsis* systematics of the Palaearctic region, and invaluable help to the first author.

### Key to species (males only) of the subgenus *Pediopsoides (Pediopsoides)*

Male *Pediopsoides (Pediopsoides) formosanus* (Matsumura) is not known, hence not included in the key. The present key is modified from Li et al. 2012.

**Table:** 

1	Aedeagal shaft with processes	2
–	Aedeagal shaft without processes (Figs 11–12, 19–20)	4
2	Aedeagal shaft with apical processes laterally directed and on either side of gonopore	*Pediopsoides (Pediopsoides) jingdongensis* Zhang
–	Aedeagal shaft with subapical processes directed dorsally or ventrally	3
3	Aedeagal shaft processes directed dorsally	*Pediopsoides (Pediopsoides) kodaiana* Viraktamath
–	Aedeagal shaft processes directed ventrally	*Pediopsoides (Pediopsoides) femorata* (Hamilton)
4	Aedeagal shaft inflated subapically in lateral view	*Pediopsoides (Pediopsoides) satsumensis* (Matsumura)
–	Aedeagal shaft either of uniform width (Fig. 11) or narrowed subapically (Fig. 19)	5
5	Clypellus black	*Pediopsoides (Pediopsoides) nigorolabium* Li, Dai & Li
–	Clypellus not black	6
6	Aedeagal shaft of uniform width in lateral view (Fig. 11); dorsal connective S-shaped (Fig. 13)	*Pediopsoides (Pediopsoides) dainghanensis* Li, Dai & Li sp. n.
–	Aedeagal shaft tapered variously distally (Fig. 19)	7
7	Style with subapical tooth-like process (Fig. 16)	*Pediopsoides (Pediopsoides) tishetshkini* Li, Dai & Li sp. n.
–	Style without a subapical tooth-like process	*Pediopsoides (Pediopsoides) bispinata* Li, Dai & Li

## Supplementary Material

XML Treatment for Pediopsoides (Pediopsoides)

XML Treatment for Pediopsoides (Pediopsoides) damingshanensis

XML Treatment for Pediopsoides (Pediopsoides) tishetshkini
